# Sleep, aging, and lifespan in *Drosophila*

**DOI:** 10.1186/1471-2202-11-56

**Published:** 2010-04-29

**Authors:** Daniel Bushey, Kimberly A Hughes, Giulio Tononi, Chiara Cirelli

**Affiliations:** 1 Dept. of Psychiatry, University of Wisconsin/Madison, Madison, Wisconsin, 53719, USA; 2 Dept. of Biological Science, Florida State University, Tallahassee, FL 32306, USA

## Abstract

**Background:**

Epidemiological studies in humans suggest that a decrease in daily sleep duration is associated with reduced lifespan, but this issue remains controversial. Other studies in humans also show that both sleep quantity and sleep quality decrease with age. *Drosophila melanogaster *is a useful model to study aging and sleep, and inheriting mutations affecting the potassium current Shaker results in flies that sleep less and have a shorter lifespan. However, whether the link between short sleep and reduced longevity exists also in wild-type flies is unknown. Similarly, it is unknown whether such a link depends on sleep amount per se, rather than on other factors such as waking activity. Also, sleep quality has been shown to decrease in old flies, but it remains unclear whether aging-related sleep fragmentation is a generalized phenomenon.

**Results:**

We compared 3 short sleeping mutant lines (*Hk*^1^, *Hk*^*Y *^and *Hk*^2^) carrying a mutation in Hyperkinetic, which codes for the beta subunit of the Shaker channel, to wild-type siblings throughout their entire lifespan (all flies kept at 20°C). *Hk*^1 ^and *Hk*^*Y *^mutants were short sleeping relative to wild-type controls from day 3 after eclosure, and *Hk*^2 ^flies became short sleepers about two weeks later. All 3 *Hk *mutant lines had reduced lifespan relative to wild-type flies. Total sleep time showed a trend to increase in all lines with age, but the effect was most pronounced in *Hk*^1 ^and *Hk*^*Y *^flies. In both mutant and wild-type lines sleep quality did not decay with age, but the strong preference for sleep at night declined starting in "middle age". Using Cox regression analysis we found that in *Hk*^1 ^and *Hk*^*Y *^mutants and their control lines there was a negative relationship between total sleep amount during the first 2 and 4 weeks of age and hazard (individual risk of death), while no association was found in *Hk*^2 ^flies and their wild-type controls. *Hk*^1 ^and *Hk*^*Y *^mutants and their control lines also showed an association between total daily wake activity over the first 2 and 4 weeks of age and hazard. However, when both sleep duration and wake activity were used in the same regression, the effects of activity were much reduced, while most of the sleep effects remained significant. Finally, *Hk*^1 ^flies and wild-type siblings were also tested at 25°C, and results were similar to those at 20°C. Namely, *Hk*^1 ^mutants were short sleeping, hyperactive, and short lived relative to controls, and sleep quality in both groups did not decrease with age.

**Conclusions:**

Different *Hk *mutations affect the sleep phenotype, and do so in an age-dependent manner. In 4 of the 6 lines tested sleep associates significantly with lifespan variation even after any effect of activity is removed, but activity does not associate significantly with lifespan after the effects of sleep are removed. Thus, in addition to environmental factors and genetic background, sleep may also affect longevity. Sleep quality does not necessarily decay as flies age, suggesting that aging-related sleep fragmentation may also depend on many factors, including genetic background and rearing conditions.

## Background

Epidemiological studies suggest that both a decrease and an increase in sleep duration are associated with reduced lifespan [1-10]. The issue of whether sleep duration affects longevity, however, remains highly controversial. In some cases an association was not found (reviewed in [11]), and most studies asked subjects about their sleep pattern only once, and did not control for work time, which is reciprocally related to sleep time [12]. Some authors, on the other hand, have argued that epidemiological studies have underestimated the mortality risk associated with short or long sleep, because they control for co-morbidities even when they should not. For instance, if short sleep causes heart disease, controlling for a history of heart disease might obscure the underlying effect of sleep duration [3,13]. Perhaps the biggest limitation of all epidemiological studies, however, is the fact that they are based on self-reported sleep duration, whose validity remains elusive [14]. Ideally, sleep should be objectively measured in hundreds or thousand of subjects at multiple time points throughout their life, but these studies are expensive and time consuming to conduct in humans.

*Drosophila melanogaster *is a powerful model to study lifespan and aging [15,16]. Flies were used to prove for the first time that lifespan is an inherited trait and that it can be extended by selection [15]. They were also instrumental to test evolutionary and mechanistic theories of aging (reviewed in [17]), clarify how dietary restriction affects lifespan [18], and identify genes that increase longevity [15,16]. Flies are also good models to study sleep [19,20], because of the striking similarities between fly and mammalian sleep [21-24]. As in mammals, sleep in flies consists of periods of sustained quiescence associated with an increased arousal threshold, is modulated by stimulants and hypnotics, is associated with changes in brain activity, and is characterized by changes in the expression of hundreds of genes [21-24]. Moreover, flies like humans sleep mostly during the night, and when sleep deprived show reduced vigilance and are less capable of performing certain tasks. Also, daily sleep amount is highest in both humans and flies during periods when the nervous system is developing, and in flies, mutations that affect this developmental plasticity also alter sleep [25]. Finally, sleep in flies, like in mammals, becomes longer and less fragmented after sleep deprivation [21-24].

We recently isolated *minisleep*, a fly line carrying a mutation in *Shaker*, a gene coding for the alpha subunit of a voltage-dependent potassium channel. *Sh*^*mns *^flies sleep only 3-4 hours/day, while their wild-type controls sleep 8-14 hours/day [26]. After crossing out genetic modifiers, we found that other *Sh *alleles, such as *Sh*^102 ^and *Sh*^*M*^, also became short sleepers. Moreover, *Sh*^*mns *^flies have reduced lifespan as compared to the control strain, and outcrossed *Sh*^102 ^and *Sh*^*M *^short sleeping flies also die earlier compared to their non-short sleeping siblings or the original normal sleeping *Sh *stocks [26]. However, whether the reduced longevity is due to short sleep, rather than to other factors such as hyperactivity, remains unclear. *Shaker *is highly conserved across species, and mice lacking *Kcna2*, which codes for the alpha subunit of a Shaker-like voltage-dependent potassium channel (a.k.a. Kv1.2), also have reduced NREM sleep and die early [27].

*Hyperkinetic (Hk) *codes for the beta (modulatory) subunit of Shaker and strong hypomorph *Hk *mutations (*Hk*^1 ^and *Hk*^*Y*^), like loss of function *Sh *mutations, cause reduced sleep [28]. Another mutation recently identified in flies, *Sleepless*, also reduces sleep, to only ~ 2 hours a day (~ 85% less than controls), as well as lifespan [29]. *Sleepless *codes for a glycosyl-phosphatidylinositol-anchored protein with unknown function, and has no obvious vertebrate homolog. *Quiver*, however, a previously identified mutation that affects the Shaker (I_A_) current, is an allele of *Sleepless*, and *Sleepless *flies have reduced levels of Shaker protein [29]. Moreover, recent data show that the *Sleepless *short sleeping phenotype is at least in part mediated by the *Shaker *current [30]. *Fumin *(sleepless in Japanese), on the other hand, a mutation in the gene coding for the *Drosophila *dopamine transporter, decreases daily sleep amount by ~ 60% but may not affect lifespan [31]. Thus, it remains unclear whether the link between reduced longevity and short sleep in flies is specific to mutations affecting the Shaker current, or is a more generalized phenomenon.

In healthy humans aging is associated with a decrease in total sleep time and in sleep efficiency (the number of awakenings after sleep onset increases), although between elderly (60-70 years) and older elderly (>70) there is no change except in sleep efficiency [32]. A recent study found increased sleep fragmentation in old flies [33], but whether sleep quality always decreases with aging in *Drosophila *remains unclear.

Here we tested whether daily sleep amount is correlated with lifespan in 3 *Hk *mutant lines, as well as in their wild-type siblings in which the Shaker current is normal. Moreover, in the same fly lines, we looked at the effects of aging on sleep quantity and quality.

## Methods

### Genetics

Genetic background profoundly affects lifespan in *Drosophila *[34], and *Sh *stocks accumulate genetic modifiers that suppress the short sleeping phenotype [26]. Thus, we always compared outcrossed males that inherited the *Hk *allele (the strong hypomorph *Hk*^1 ^and *Hk*^*Y *^or the weak hypomorph *Hk*^2^) to siblings that inherited a wild-type allele, because siblings have a common genetic background and an equal chance of inheriting modifiers. *Hk *alleles were outcrossed by mating females from *Hk *stocks to Canton-S (CS) males. Virgin heterozygous females were then collected and crossed again to CS males. Since *Hk *is on the X chromosome, the female progeny can inherit either the wild-type or a mutant *Hk *allele. Heterozygous females were used to continue the outcrossing, but could not be distinguished from homozygous wild-type females because the leg shaking phenotype caused by *Hk *mutations is recessive. We therefore setup multiple crosses between single females and CS males, and kept only those that produced ether-induced leg shaking male progeny. Crosses with homozygous wild-type females did not produce ether-induced leg shaking males and were discarded. After 4-5 generations of outcrossing mutants and wild-type siblings were collected for testing.

### Sleep analysis

Flies (n ≥ 199/line) were cultured and tested at 20°C, 68% humidity, 12 h:12 h light:dark cycle (lights on at 8 am), on yeast, dark corn syrup and agar food. These experimental conditions were selected because they had been previously used in our laboratory for an extensive analysis of sleep quantity and quality [20,26,35]. Stocks were expanded in half-pint bottles under controlled standard larval densities [36]. Within 24-48 hours after eclosure mutants were separated from wild-type siblings using the ether-induced leg shaking phenotype caused by *Hk *mutations. Each individual fly was housed in glass tubes (5 mm diameter by 65 mm length; one fly per tube) with enough food for 1 week. Locomotor activity in the tube was recorded using the *Drosophila *Activity Monitor System (DAMS, Trikinetics, Waltham, MA). Specifically, as each fly moves back and forth in the tube, it interrupts an infrared light beam that bisects the tube. Each crossing is counted as a movement and the number of beam crossings was counted over 1-min periods. Every week until death each fly was transferred without anesthesia to a new glass tube. Thus, both sleep and lifespan were recorded simultaneously from the same set of flies. DAMS monitors were housed inside environmental chambers where temperature and humidity were kept constant. Values from 24-hour recordings were analyzed daily using custom-designed software developed in our laboratory and based on Statistica (StatSoft; [35]). The data were further analyzed using Matlab (Mathworks; [35]) and SAS/STAT^® ^software v.9.1. Based on previous work [20,26,35], sleep was defined as any period of uninterrupted behavioral immobility (0 counts/min) lasting > 5 min, because such periods are associated with an increased arousal threshold, which distinguishes sleep from quiet waking. If the 5-min period had one or more activity counts, then the interval was counted as a wake period. Brief awakenings were defined as 1-min epochs with 1 beam crossing (1 count) preceded and followed by sleep (>5 min with no counts). Flies were considered dead if they showed no activity for 24 hours, and death in each fly was confirmed by visual inspection. The last 24 hours of life were excluded from both sleep and lifespan analysis, to rule out effects due to death-imminent processes. Flies that were found stuck in the food were also excluded from the analysis.

In a different experiment *Hk*^1 ^flies and wild-type siblings were raised and tested at 25°C. Experimental conditions were identical to those used at 20°C (68% humidity, 12 h:12 h light:dark cycle, lights on at 8 am), except that flies were transferred to fresh tubes every 5 days rather than 7 days.

### Analysis of lifespan

As a first step in data analysis, non-parametric log rank tests of the cumulative survival curves and estimations of mean/median lifespan were performed using SAS Proc Lifetest. This survival analysis compares survival among populations based on the distribution of ages at death, does not require fitting a specific parametric model, and incorporates any censored data (e.g., flies escaped during the weekly transfer to fresh tubes; in all experiments, these flies accounted for <5% of all flies). To test for significance, both log-rank and Wilcoxon tests were used. Since these tests were always consistent, only the log-rank tests are reported.

To test the effects of sleep parameters on lifespan, we used Cox regression models, as implemented in SAS Proc Phreg. The procedure uses maximum likelihood estimation to test the effects of individual-level covariates (predictor variables) on the probability of survival at different ages [37]. More specifically, the procedure implements a partial likelihood method to estimate coefficients of a regression model relating an individual's *hazard *(the instantaneous risk of death at any given time) to a set of covariates measured on the same individual. The procedure also provides tests of significance for these coefficients, using a standard maximum likelihood ratio chi-square statistic. These coefficients can be interpreted as one interprets the coefficients of a linear regression model: a positive coefficient indicates that the hazard tends to increase with increasing values of the covariate, and a negative coefficient indicates that the hazard decreases with increasing values of the covariate. Multiple covariates can be accommodated in a single analysis. In that case, the coefficient for each covariate represents the association between that covariate and the hazard, after removing effects of all other covariates, analogous to a partial regression coefficient in multiple regression. We chose this procedure because it does not require assumptions about the form of the survival distribution, it provides a flexible method to test the effects on survival patterns of multiple covariates simultaneously, and it accommodates censored data. The proportional hazards assumptions of the model were tested using standard procedures [37].

## Results

### Hypomorphic mutations in the Hk gene decrease sleep, increase locomotor activity, and reduce lifespan

**Figure 1:**
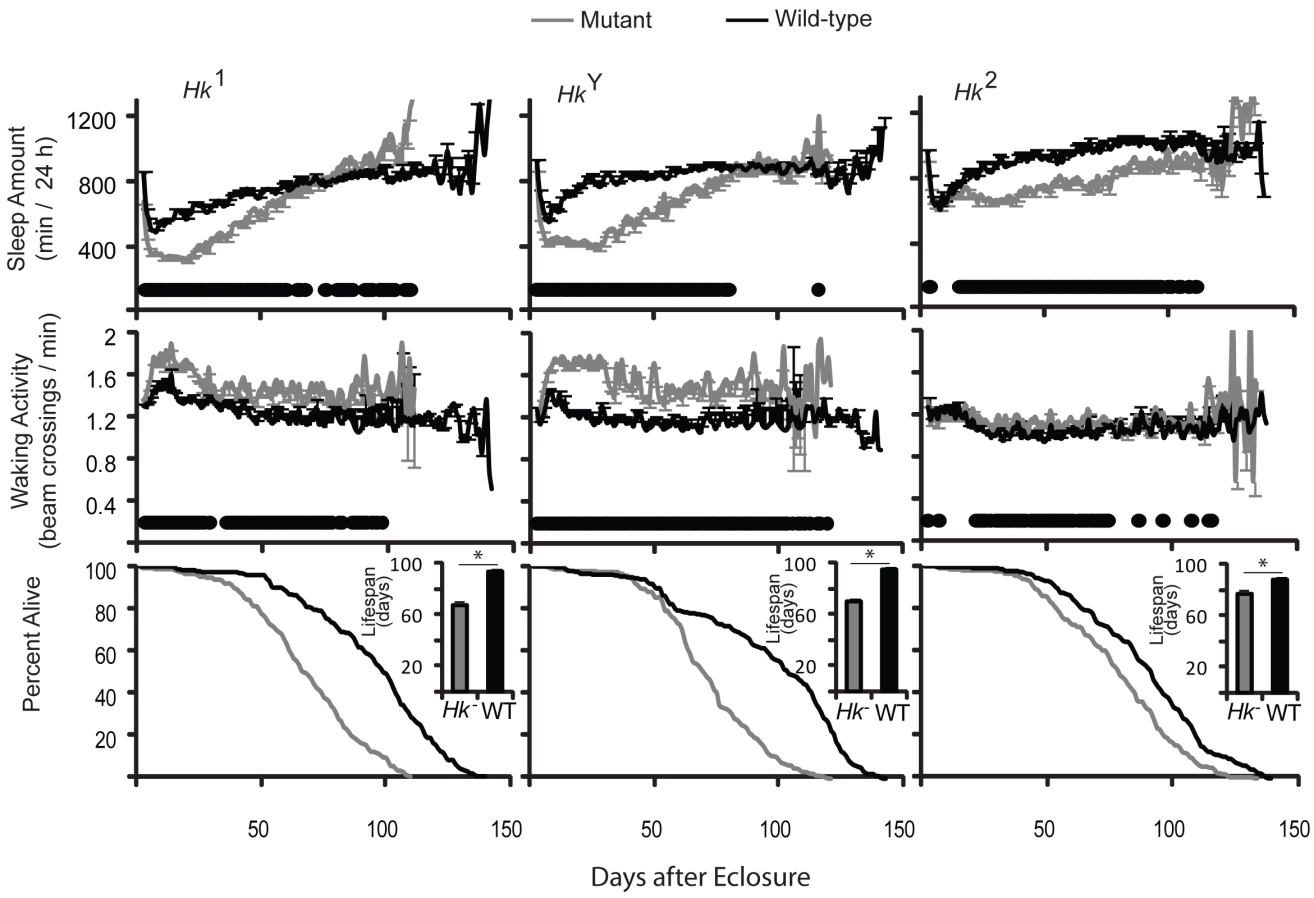
**Sleep duration and lifespan are reduced in *Hk *mutants, while waking activity is increased**. Twenty-four hour values of sleep and waking activity from eclosure to death and survival curves in male *Hk *mutants and wild-type siblings. Inset. Average lifespan. Number of flies: *Hk*^1 ^= 220; siblings *Hk*^+ ^= 219; *Hk*^*Y *^= 226; siblings *Hk*^+ ^= 231; *Hk*^2 ^= 199; siblings *Hk*^+ ^= 216. Black dots and asterisks indicate a significant difference from wild-type (p < 0.05, Mann-Whitney test for sleep analysis; p < 0.0001, log-rank test for lifespan analysis). Values are mean ± SEM.

Fig. 1 shows daily sleep amount and waking activity for 3 *Hk *mutant lines and their wild-type siblings (all males) throughout their entire lifespan. Both *Hk*^1 ^and *Hk*^*Y *^flies slept less than controls at day 3 and the severity of the short sleeping phenotype increased after the first week, peaking at day 18 in *Hk*^1 ^flies (-45%; mutant - wild-type/wildtype × 100), and at day 25 in *Hk*^*Y *^mutants (-51%). As the flies aged the difference in sleep duration decreased, although was still significant at day 76 in *Hk*^*Y *^flies, while *Hk*^1 ^mutants were no longer consistently short sleepers after day 60. In *Hk*^2 ^mutants, instead, the short sleep phenotype appeared only after day 16, peaked at day 32 (-32%), and persisted until day 95.

Waking activity was not significantly affected by age in *Hk*^1 ^and *Hk*^*Y *^flies, which remained hyperactive relative to their wild-type siblings from day 2 until death (Fig. 1). In contrast, *Hk*^2 ^mutants were consistently hyperactive from day 20 until day 74 (Fig. 1). Thus, all *Hk *mutants were short sleeper and hyperactive for at least half of their life, but the 2 phenotypes did not necessarily occur at the same time. Specifically, late in life *Hk*^1 ^and *Hk*^*Y *^flies were still hyperactive but no longer short sleeper, while old *Hk*^2 ^flies were still sleeping less than controls despite showing less hyperactivity.

As shown in the survival curves of Fig. 1, both mutants and wild-type flies showed a lag period before going into an exponential death phase. In all 3 mutant lines the death rate during the exponential death phase was increased relative to their wild-type siblings, resulting in a decrease in lifespan of -29%, -26%, and -13% in *Hk*^1^, *Hk*^*Y *^and *Hk*^2 ^flies, respectively (p < 0.0001, log-rank test; Fig. 1, inset). Thus, *Hk*^1^, *Hk*^*Y *^and *Hk*^2 ^flies lived respectively 27, 24, and 11 days less than their wild-type siblings.

### Changes in sleep with aging in all flies

**Figure 2:**
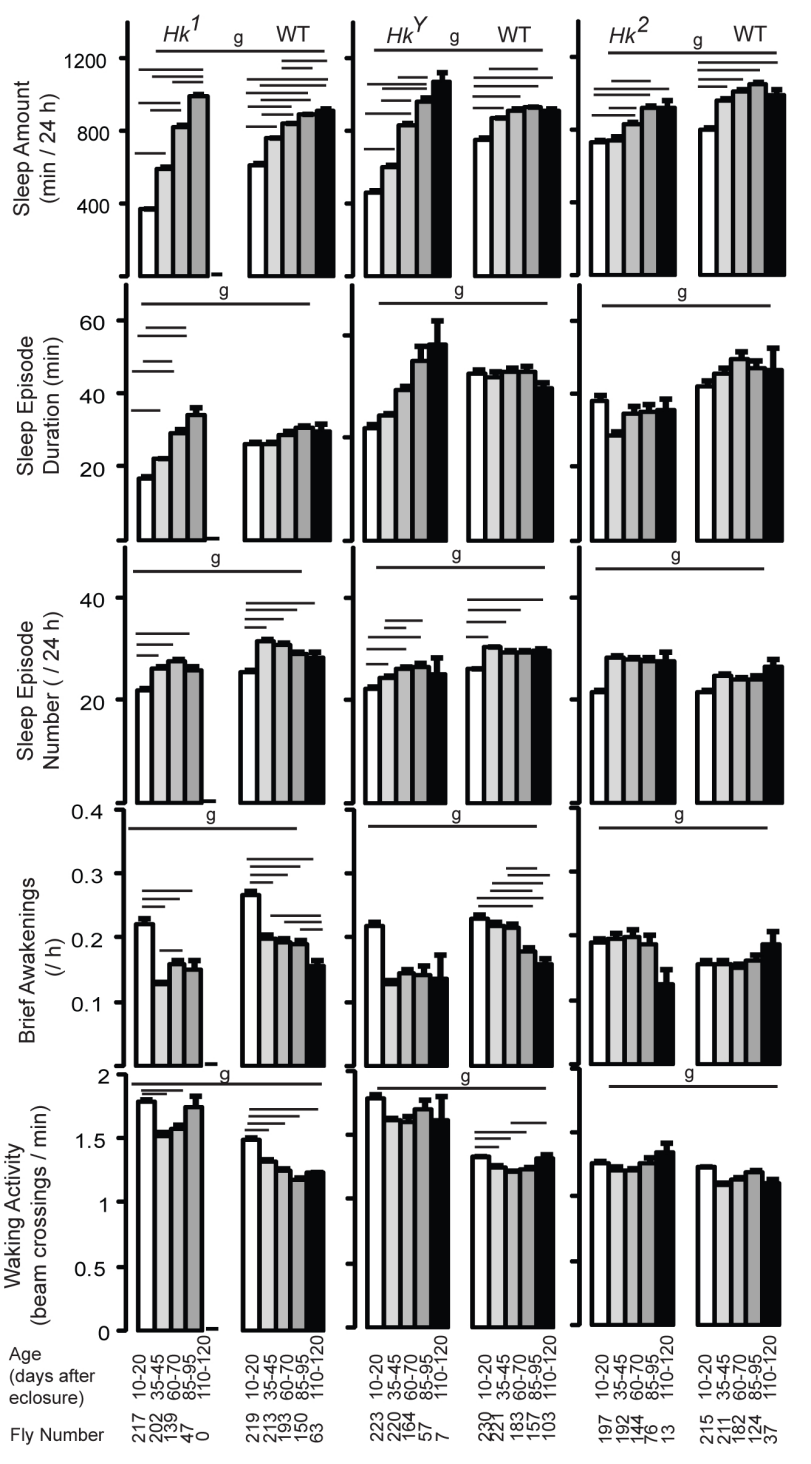
**Sleep parameters and waking activity at different ages in all flies**. For each age range (from 10 to 120 days; shown below each bar) mean values (+ SEM) were averaged across eleven 24-hour intervals per each fly, and then averaged across all flies. The number of flies tested at each age is indicated below each bar. Genotype (g) effects were tested across all ages (Kruskal-Wallis test, p < 0.05). Horizontal bars indicate significant age-related effects within each genotype (Friedman test followed by Wilcoxon signed-rank test, p < 0.05).

To determine how aging affects sleep quantity and sleep quality we averaged waking activity, daily sleep amount, number of sleep episodes, mean duration of sleep episodes, and number of brief awakenings across different 11-day periods, from day 10 until day 120 after eclosure (Fig. 2). We specifically left out the first 9 days after eclosure, when profound developmental changes are known to affect sleep. For instance, we have shown that the loss of the developmentally regulated gene *dFmr1 *is necessary for the dramatic drop in sleep time during the first 3 days after eclosure ([25]; Fig. 1).

In all lines, sleep amount increased with age, an effect most pronounced in *Hk*^1 ^and *Hk*^*Y *^mutants (Fig. 2). Sleep episode duration increased only in *Hk*^1 ^flies, while the number of sleep episodes increased in most lines early in life (day 10-45), but remained constant afterwards. The number of brief awakenings either decreased or did not change with age. Overall these data suggest that sleep quality does not significantly change in flies in the second part of their life. Moreover, waking activity remained constant and did not decline with age, the only exception being *Hk*^1 ^wild-type siblings, which were more active at day 10-20 compared to all other periods (Fig. 2).

**Figure 3:**
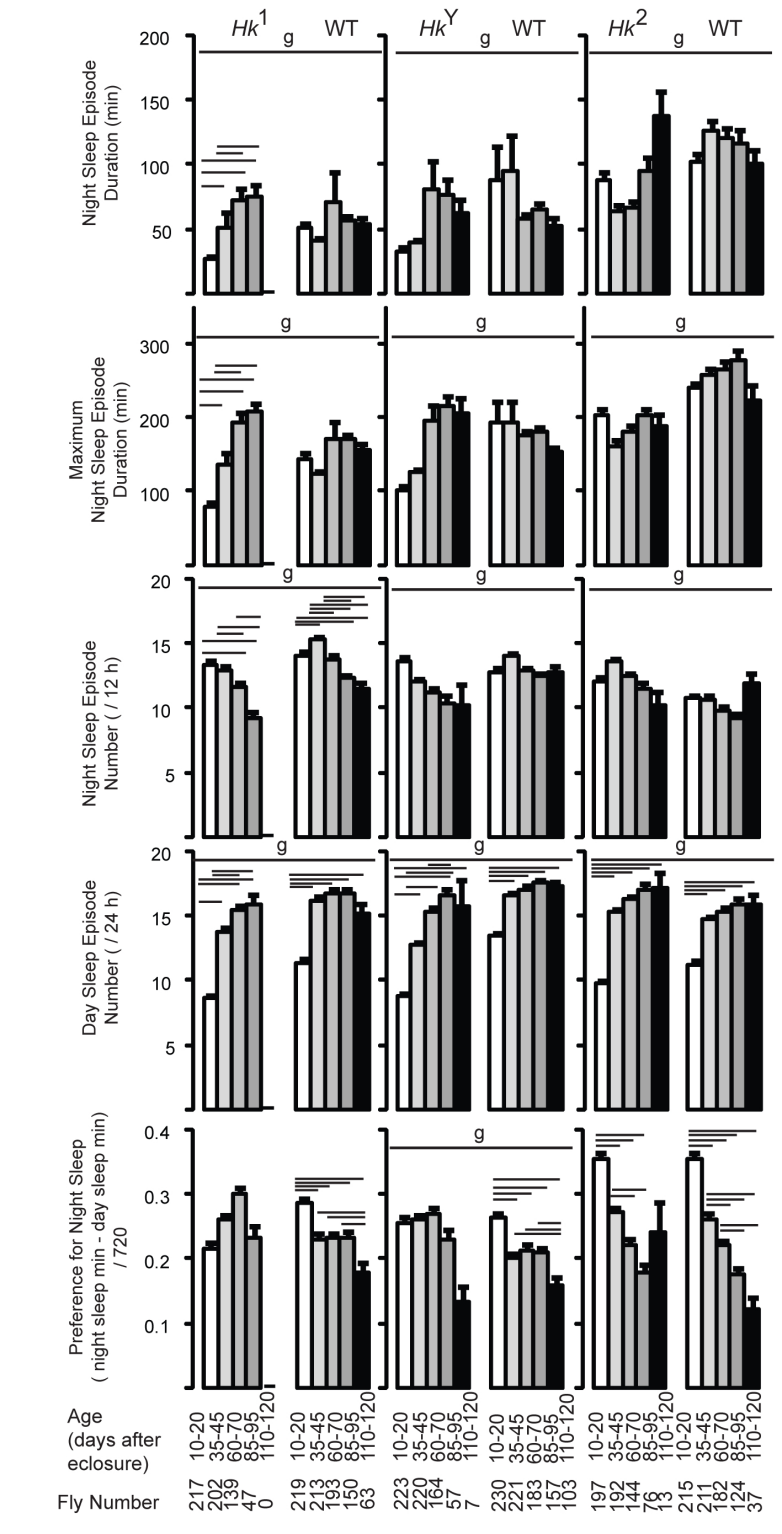
**Sleep parameters during night and day in all flies**. Values are mean + SEM (eleven 12-hour intervals averaged per each fly, and then averaged across all flies). The number of flies tested at each age is shown below each bar. Genotype (g) effects were tested across all ages (Kruskal-Wallis test, p < 0.05). Horizontal bars indicate significant age-related effects within each genotype (Friedman test followed by Wilcoxon signed-rank test, p < 0.05).

**Figure 4:**
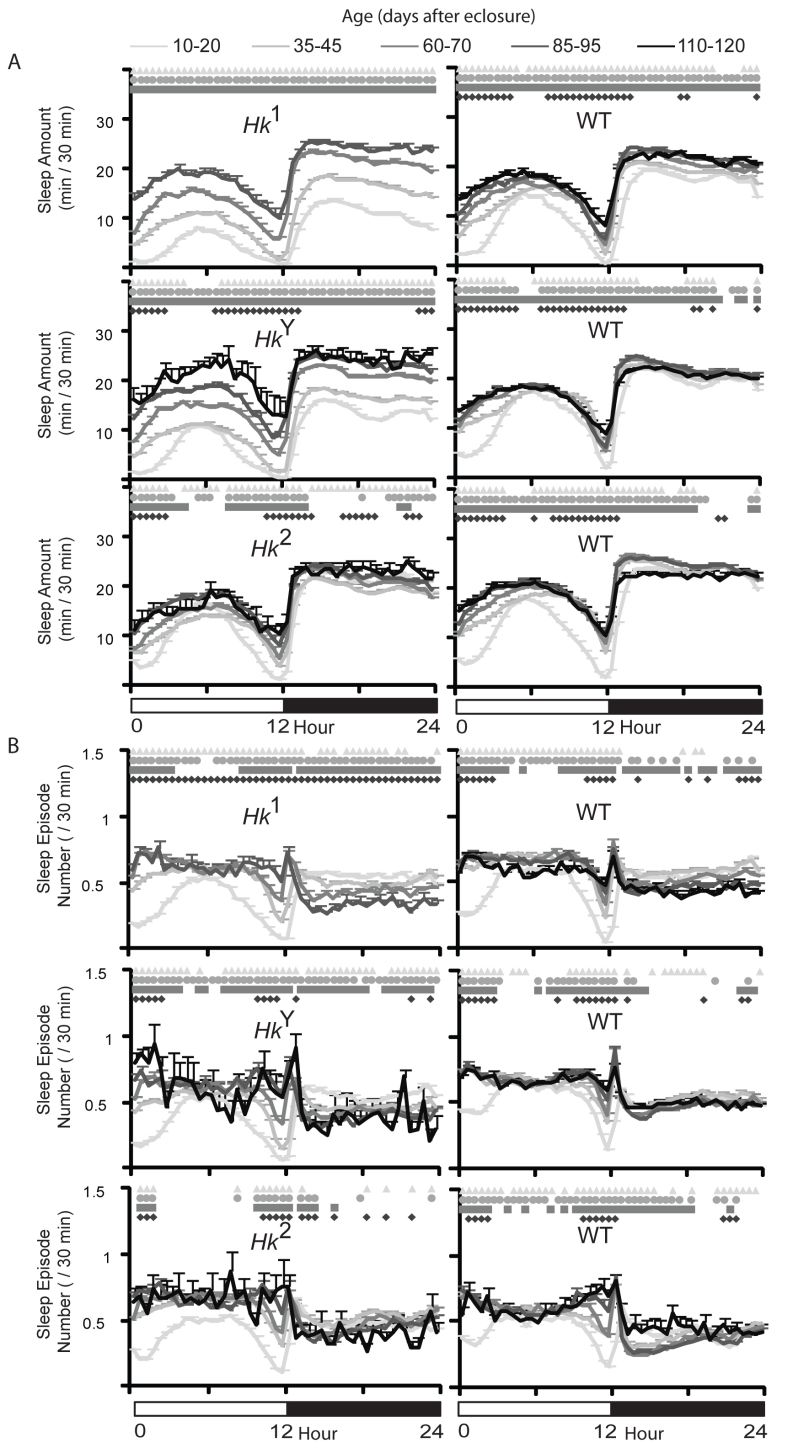
**Time course of sleep parameters in all flies**. Values are mean + SEM (eleven 30-min intervals averaged per each fly, and then averaged across all flies). Comparisons (Friedman test followed by Wilcoxon signed-rank test, p < 0.05) are between the 10-20 day period and all following ages: triangle (vs 35-45), circle (vs 60-70), square (vs 85-95), diamond (vs 110-120). Data are derived from the same flies described in Figures 2 and 3.

Since sleep occurs primarily at night we also measured sleep parameters specifically during the 12-hour dark period. Consistent with the 24-hour results, sleep amount increased with age in all lines (not shown), and the mean episode duration increased in *Hk*^1 ^mutants but not in the other lines (Fig. 3), and so did the maximal duration of sleep episodes (Fig. 3). In most flies however, the number of sleep bouts at night showed a trend to decrease with age, an effect contrasted by the increase in the number of sleep episodes during the day in all lines (Fig. 3). This suggested that older flies may be sleeping more during the day, a result confirmed by measuring the preference for sleep at night, which decreased with age in all lines except *Hk*^1 ^and *Hk*^*Y *^(Fig. 3), and by looking at the sleep time course throughout the 24 hours (Fig. 4A). In *Hk*^1 ^and *Hk*^*Y *^mutants sleep time increased with age during both day and night, while in *Hk*^2 ^mutants and wild-type lines sleep bouts became more frequent at the beginning and the end of the light phase, when young flies are normally most active (Fig. 4B). Overall, these results show that older flies sleep more at the light-dark and dark-light transitions.

### Changes in sleep with aging in the 70--day cohort

In the previous analysis all flies were included, which could be problematic for several reasons. First, sick flies that died prematurely may have affected the results, since human studies show that the effects of age on sleep vary depending on whether or not only healthy subjects are included in the study [38]. Second, if survival is linked to certain sleep parameters, the latter will be overrepresented in very old subjects. Third, very few flies survived after 90-100 days, which means that in some cases (*Hk*^*Y*^) as few as 7 flies were used for statistics. We thus repeated the analysis using only flies that were still alive at day 70 and did not die at least until day 73. The limit of 70 days was selected because it is the average lifespan in the shortest living mutants, *Hk*^1 ^and *Hk*^*Y*^, so at least 50% of the flies in all lines survive to this age. Thus, the 70-day cohort likely contains mostly healthy flies, and is large enough to allow statistically meaningful comparisons.

**Figure 5:**
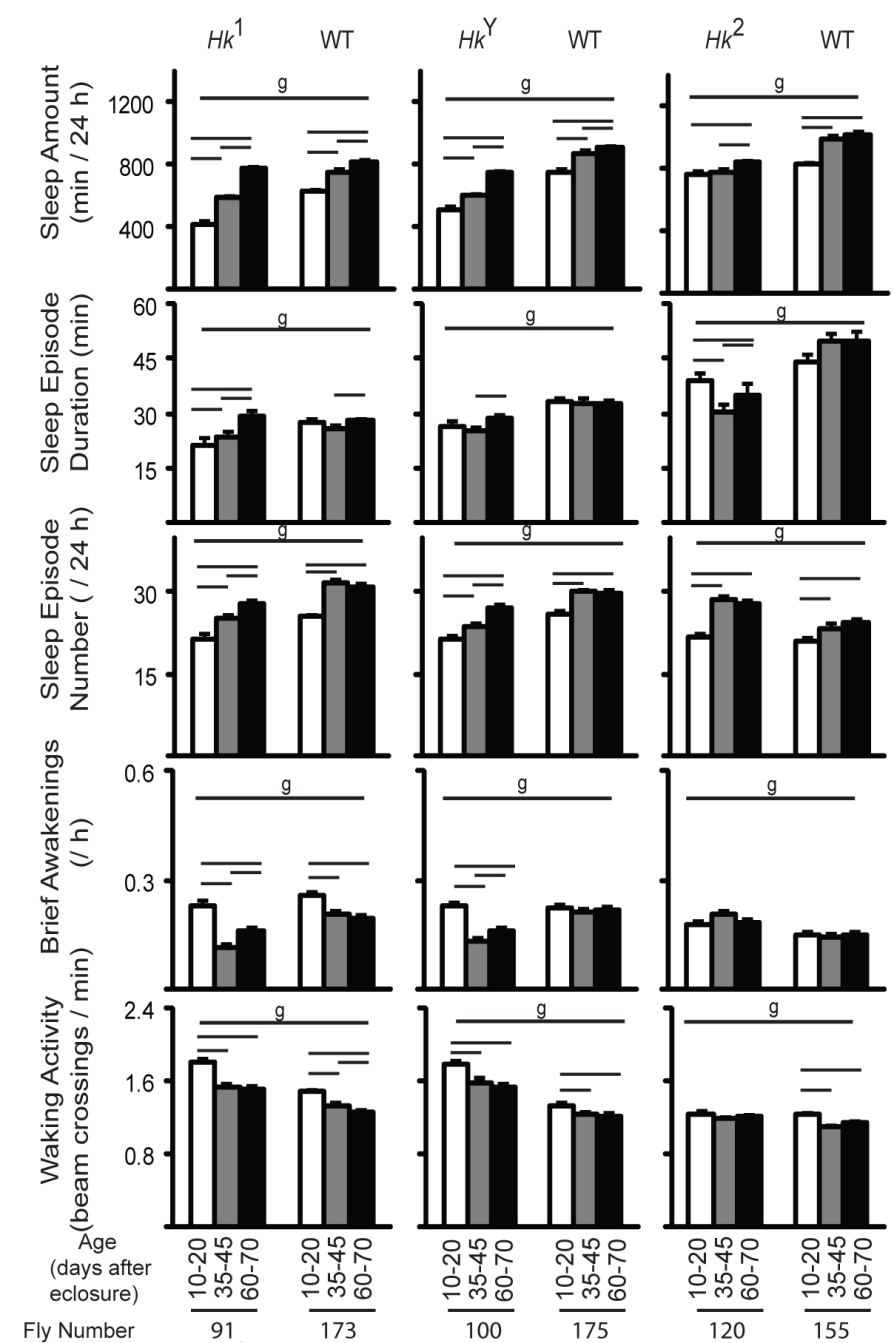
**Sleep parameters and waking activity at different ages in flies that lived at least 73 days**. Values are mean + SEM (eleven 24-hour intervals averaged per each fly, and then averaged across all flies). Genotype (g) effects were tested across all ages (Kruskal-Wallis test, p < 0.05). Horizontal bars indicate significant age-related effects within each genotype (Friedman test followed by Wilcoxon signed-rank test, p < 0.05).

As before we focused first on 24-hour values in young (average of days 10-20), "middle age" (days 35-45), and old (days 60-70) flies. Across age, all sleep parameters showed significant differences due to genotype (Kruskal-Wallis test, p < 0.05; Fig. 5). Widespread differences due to age were also found within each line (Friedman test followed by Wilcoxon signed-rank test, p < 0.05; Fig. 5). Consistent with the previous results using all flies, daily sleep amount increased with age in all lines, although in *Hk*^2 ^mutants the increase was small and only occurred in old age. Also in agreement with the previous results, old flies slept more mainly because the number of sleep episodes increased, and this effect occurred primarily during the light period. The average duration of sleep episode did not change in most lines, increased in *Hk*^1 ^mutants, and decreased in *Hk*^2 ^mutants. The number of brief awakenings either did not change or decreased in old flies relative to young flies. Thus, flies from 3 *Hk *mutant lines and 3 wild-type lines that aged successfully, as indicated by the fact that they were still alive at day 73, tended to sleep more with aging but did not show clear evidence of increased sleep fragmentation. Finally, the waking activity showed a modest decrease with age in all lines except in *Hk*^2^, in which it remained constant (Fig. 5).

**Figure 6:**
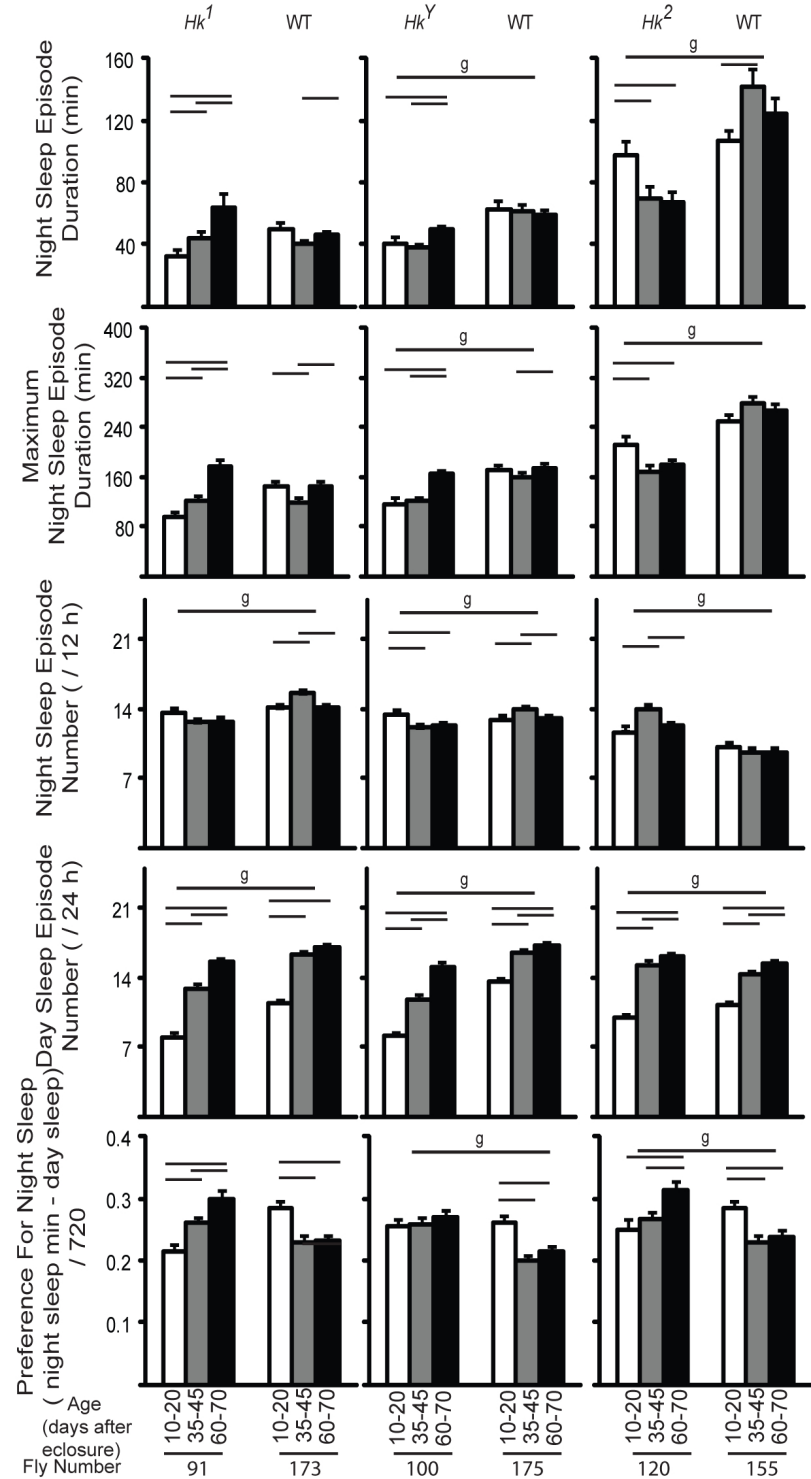
**Sleep parameters during night and day in flies that lived at least 73 days**. Values are mean + SEM (eleven 12-hour intervals averaged per each fly, and then averaged across all flies). Genotype (g) effects were tested across all ages (Kruskal-Wallis test, p < 0.05). Horizontal bars indicate significant age-related effects within each genotype (Friedman test followed by Wilcoxon signed-rank test, p < 0.05).

**Figure 7:**
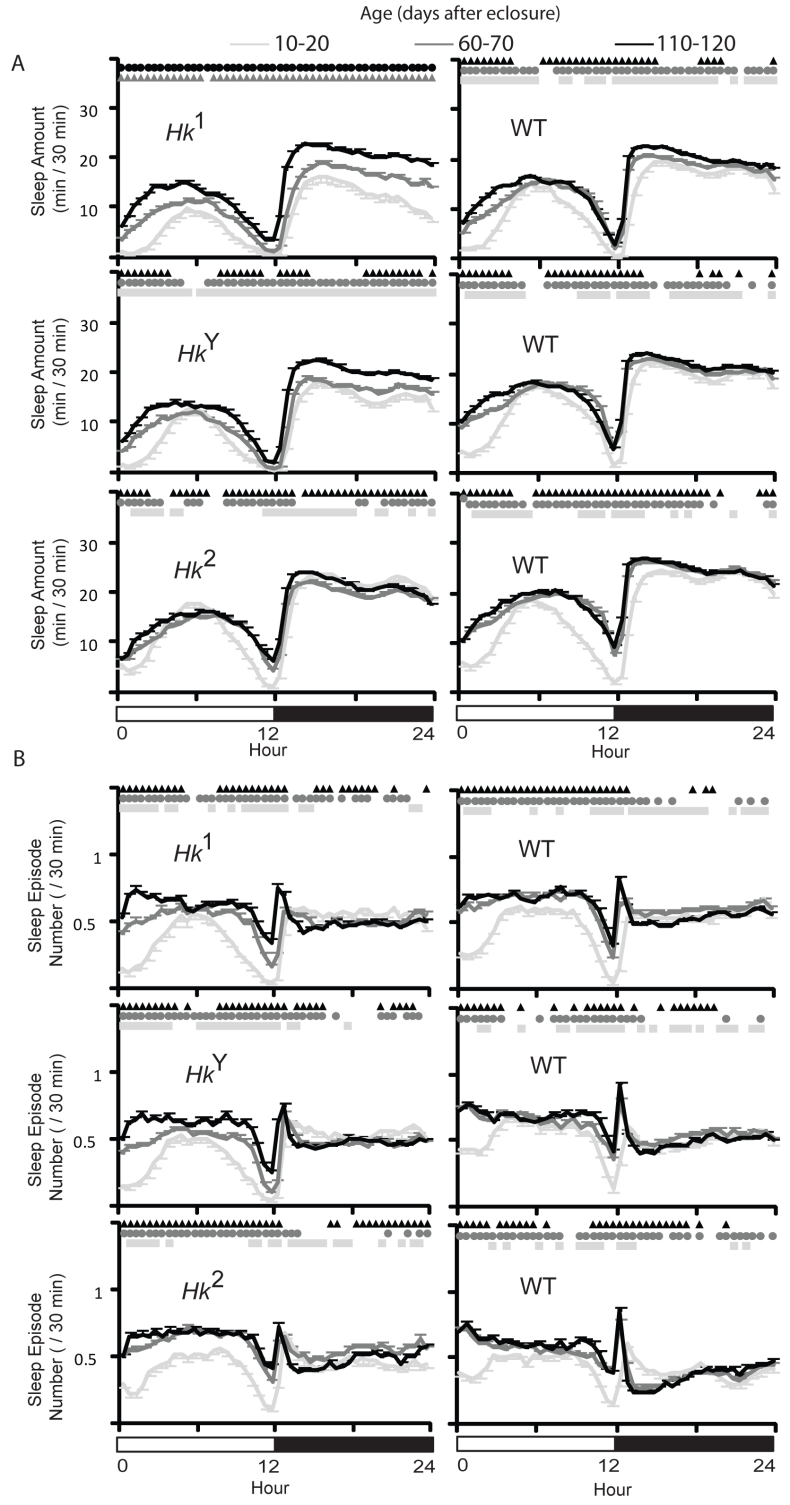
**Time course of sleep parameters in flies that lived at least 73 days**. Values are mean + SEM (eleven 30-min intervals averaged per each fly, and then averaged across all flies). Comparisons (Friedman test followed by Wilcoxon signed-rank test, p < 0.05) are between the 10-20 day period and all following ages: triangle (vs 35-45), circle (vs 60-70), square (vs 85-95), diamond (vs 110-120). Data are derived from the same flies used in Figures 5 and 6.

The mean duration of sleep bouts at night either increased or remained constant in all lines except *Hk*^2^, which showed a decrease (Fig. 6). The number of sleep episodes at night showed no consistent trend, while sleep bouts during the day became consistently more frequent with age in all lines. In mutant flies the preference for sleep at night either increased (*Hk*^1 ^and *Hk*^2^) or remained constant (*Hk*^*Y*^), while in all wild-type siblings it decreased between young and middle age, and remained constant afterwards. Analysis of the 24-hour sleep time course confirmed that with age *Hk*^1 ^and *Hk*^*Y *^flies slept more during both day and night (Fig. 7A), while wild-type siblings increased sleep amount primarily during the day. As before when using all flies, we found that the increase in sleep number was most pronounced at the light-dark and dark-light transitions (Fig. 7B). Overall, the results with the 70-day cohort are similar to those seen when all flies were included.

### Changes in sleep with aging at 25°C

**Figure 8:**
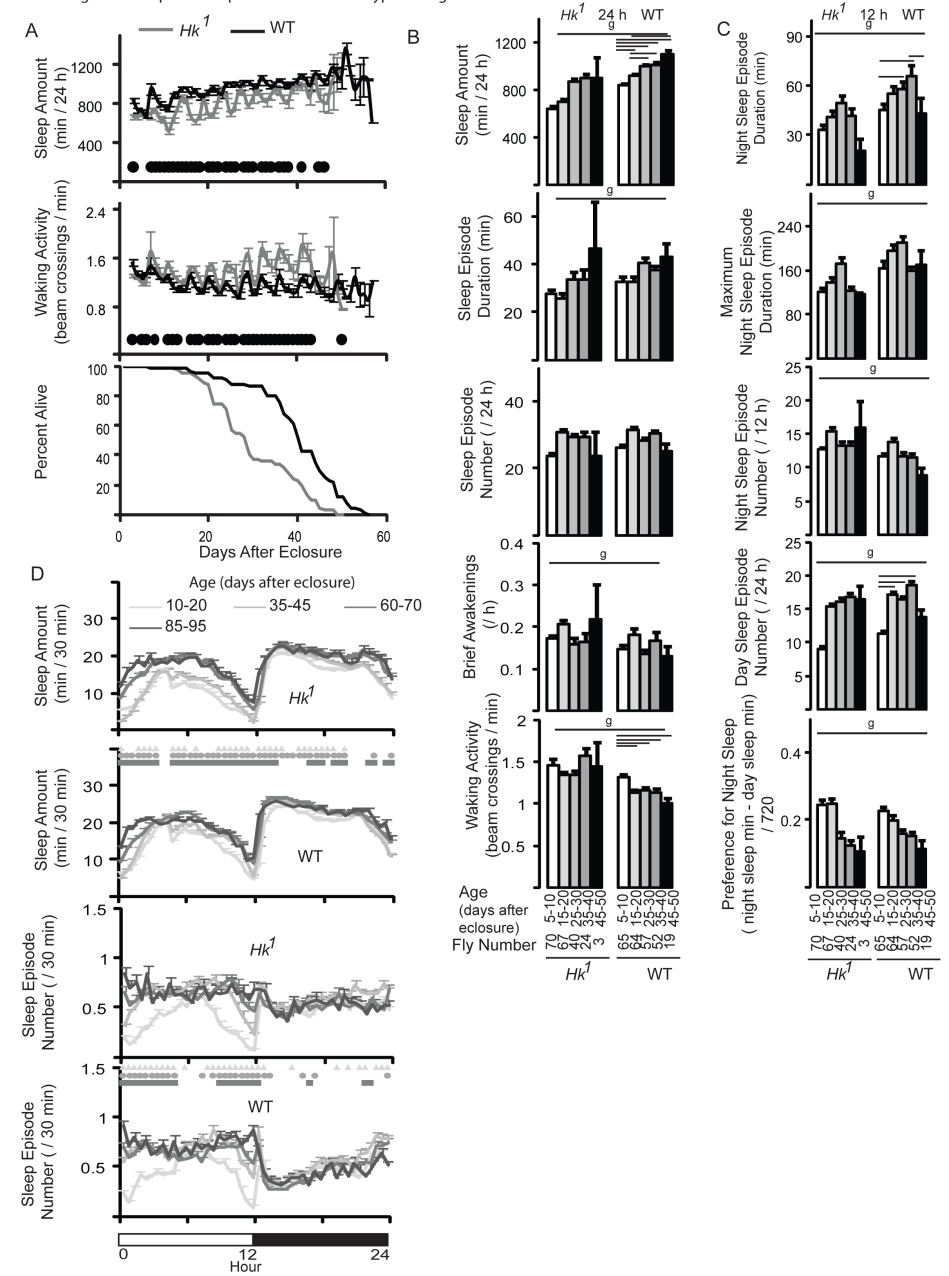
**Sleep and lifespan in *Hk*^1 ^and wild-type siblings reared and tested at 25°C**. A. Twenty-four hour values of sleep and waking activity from eclosure to death and survival curves. Circles represent significant differences (Mann-Whitney test; p < 0.05) B. Sleep parameters (24-hour values) at different ages. C. Sleep parameters during night and day. Age range and number of flies are shown below each bar. Genotype (g) effects were tested across all ages (Kruskal-Wallis test, p < 0.05). Horizontal bars indicate significant age-related effects within each genotype (Friedman test followed by Wilcoxon signed-rank test, p < 0.05). D. Time course of sleep parameters in 30-min bins. Comparisons (Friedman test followed by Wilcoxon signed-rank test, p < 0.05) are between the 10-20 day period and all following ages: triangle (vs 35-45), circle (vs 60-70), square (vs 85-95).

We also tested *Hk*^1 ^flies and wild-type siblings reared and monitored at 25°C (*Hk*^1 ^n = 70; WT n = 65). *Hk*^1 ^mutants were short sleeping and hyperactive compared to controls during most of their life (Fig. 8A), but the short sleep phenotype was not as pronounced as at 20°C (max % decrease was -34% at day 15). Mean lifespan at 25°C was ~ 50% shorter than at 20°C in both lines, and as before *Hk*^1 ^flies lived less than controls (29 vs 37 days; p < 0.0001, log-rank test).

Twenty-four hour sleep amounts increased with age in wild-type flies, while duration and number of sleep episodes and number of brief awakenings did not change (Fig. 8B). No significant changes were seen in *Hk*^1 ^flies, although a trend towards more sleep was seen in older flies (Fig. 8B).

Consistent with the results at 20°C, with age wild-type siblings increased sleep mainly during the day, and did so by increasing the number of bouts at the beginning and at the end of the light period (Fig. 8C, D). Number and duration of sleep episodes did not consistently change at night in either *Hk*^1 ^mutants or controls, suggesting that aging does not decrease the quality of night sleep at 25°C. Overall, the trends observed at 25°C were similar to those seen at 20°C, although many differences did not reach significance, likely due to fewer flies used in this experiment relative to that at 20°C.

### In *Hk*^1 ^and *Hk*^*Y *^mutants and wild--type siblings sleep amount and mortality rate are negatively correlated

**Figure 9:**
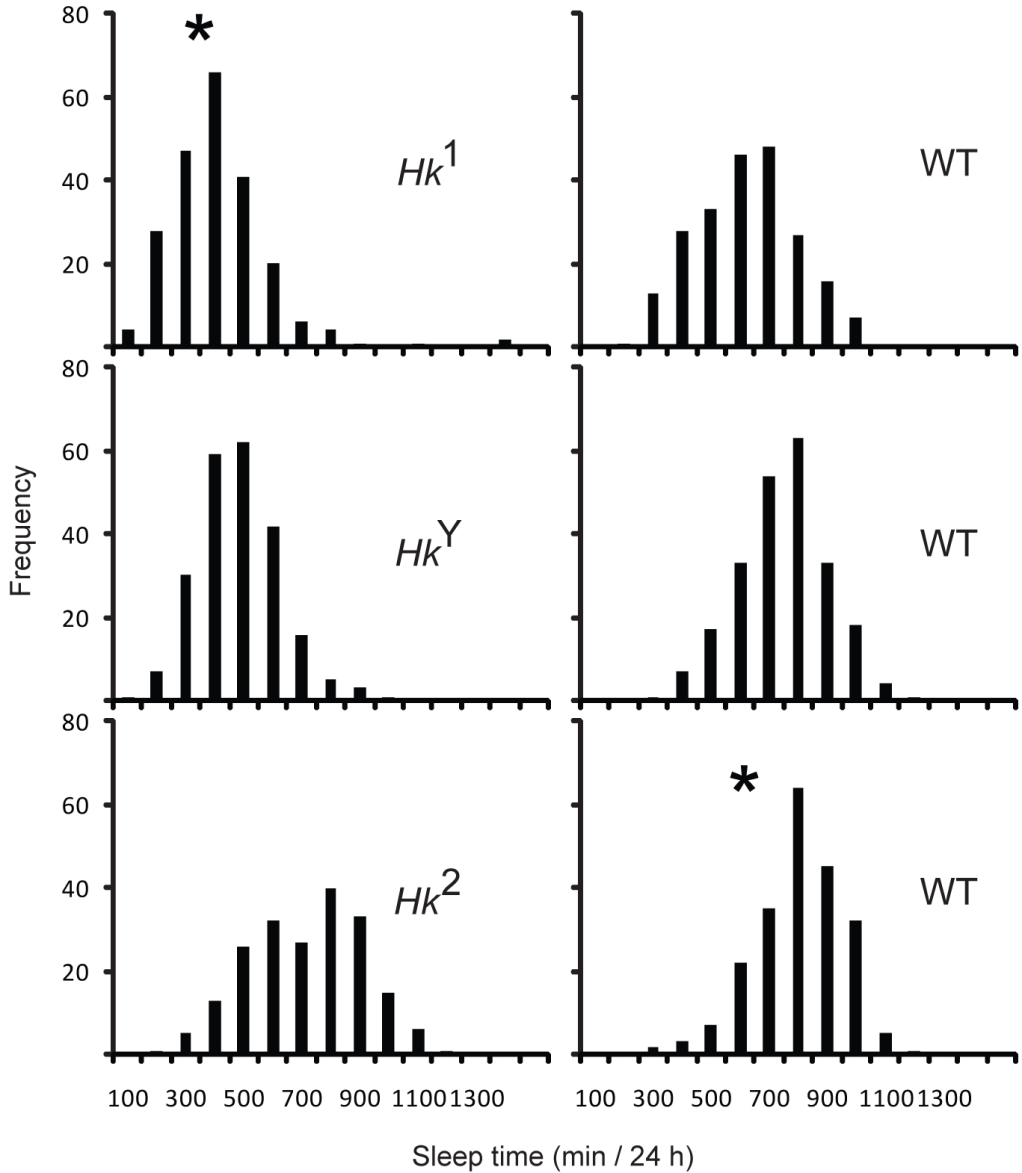
**Interindividual variability in daily sleep amount**. Mean values of daily sleep amount (min/24 h) from day 1 to day 28 per each fly (same flies as in Fig. 1). * indicates lines in which the null hypothesis (normal distribution) was rejected (p < 0.05, Lilliefors test of normality).

The data described above show that the effects on lifespan are more pronounced in the strong hypomorphic *Hk *mutants *Hk*^1 ^and *Hk*^*Y*^, which also show the strongest short sleep phenotype and the largest increase in locomotor activity, and less evident in flies carrying the weak hypomorphic *Hk*^2 ^allele, which also has smaller effects on sleep duration and waking activity. This suggests that short sleep and/or hyperactivity may decrease longevity. Consistent with previous observations [22] we also found that within each of the 6 tested lines daily sleep amount was relatively consistent from one day to the next within each fly (not shown), but highly variable and distributed almost normally across flies (Fig. 9). Of note, however, the distribution of sleep amounts in wild-type controls of *Hk*^2 ^mutants was skewed towards longer sleep relative to the wild-type controls of *Hk*^1 ^and *Hk*^*Y *^mutants (Fig. 9).

We used Cox regression analysis to test whether, within each line, there was an association between sleep duration or wake activity and the individual's hazard (the instantaneous risk of death at any given time). Daily sleep amounts were averaged across the first 2 or 4 weeks of age, because we reasoned that if sleep is important for the overall health of the fly, it should affect lifespan at an early stage of their life. We only focused on sleep quantity over 24 hours, and not on day and night sleep amounts taken separately, nor on other sleep parameters that reflect sleep quality, such as the mean or maximal duration of sleep episodes and the number of brief awakenings during sleep. This is because we found that in flies all these parameters were strongly correlated with each other. For instance, day and night sleep amounts were highly positively correlated with each other and with total (24 h) sleep amounts. The same was true for day and night sleep episode durations, which in turn were also highly positively correlated with day and night sleep amounts. The latter were strongly negatively correlated with the number of day and night brief awakenings. This high level of intercorrelation may affect the results of the Cox regression analysis, because when any two of these sleep parameters are included in the model, the effect of one (e.g. sleep episode duration) is 'corrected' for the effects of the other (e.g. total daily sleep amount). Thus, if the two sleep parameters reflect the same underlying biological process, as suggested by their high level of correlation, the results are difficult to interpret and may obscure the direct relationship between any predictor variable and mortality rate. For these reasons we focused on 24-hour sleep amount, which is also the single sleep parameter most often reported in human epidemiological studies.

The regression coefficient relating hazard to daily sleep amount, averaged across the first 2 or 4 weeks of age, was negative and highly significant in *Hk*^1 ^and *Hk*^*Y *^mutants but not in *Hk*^2 ^mutants (Table 1A), and similar results were also found in wild-type siblings of the same fly lines (Table 1B). Specifically, in wild-type controls of *Hk*^1 ^and *Hk*^*Y *^mutants there was a negative relationship between total sleep amount during the first 2 and 4 weeks of age and the risk of death, while no association was found in *Hk*^2 ^flies and their wild-type controls, although a trend was present at 4 weeks of age (*Hk*^2 ^p = 0.15, controls p = 0.08).

**Table 1:** Cox regression analysis in Hk mutants (A, C) and their wild-type siblings (B, D)

A				
Line	Days	Variable	Regression coefficient (×10^2^)	P
HkY	14 d	**Total Sleep**	**-4.32**	**<0.0001**
	28 d	**Total Sleep**	**-5.43**	**<0.0001**
				
Hk1	14 d	**Total Sleep**	**-4.94**	**<0.0001**
	28 d	**Total Sleep**	**-5.76**	**<0.0001**
				
Hk2	14 d	Total Sleep	0.855	0.259
	28 d	Total Sleep	-1.09	0.152
B				
				
Wt-HkY	14 d	**Total Sleep**	**-2.02**	**0.022**
	28 d	**Total Sleep**	**-2.06**	**0.023**
				
Wt-Hk1	14 d	**Total Sleep**	**-5.42**	**<0.0001**
	28 d	**Total Sleep**	**-5.38**	**<0.0001**
				
Wt-Hk2	14 d	Total Sleep	-1.16	0.178
	28 d	Total Sleep	-1.47	0.083
				
C				
				
HkY	14 d	**Total Activity**	**1.63**	**<0.0001**
	28 d	**Total Activity**	**1.42**	**<0.0001**
				
Hk1	14 d	**Total Activity**	**1.63**	**<0.0001**
	28 d	**Total Activity**	**1.26**	**<0.0001**
				
Hk2	14 d	Total Activity	0.454	0.289
	28 d	Total Activity	0.774	0.078
D				
				
Wt-HkY	14 d	**Total Activity**	**1.04**	**0.012**
	28 d	**Total Activity**	**1.22**	**0.011**
				
Wt-Hk1	14 d	**Total Activity**	**1.83**	**<0.0001**
	28 d	**Total Activity**	**2.13**	**<0.0001**
				
Wt-Hk2	14 d	Total Activity	0.032	0.948
	28 d	Total Activity	0.234	0.668
				

In addition to sleep, we studied mean waking activity (number of beam crossings/min awake) and total activity (sum of all beam crossings). Mean activity did not show any relationship with risk of death in any of the lines examined (not shown). On the other hand, the regression coefficient relating hazard to total activity across the first 2 or 4 weeks of age was positive and highly significant in *Hk*^1 ^and *Hk*^*Y *^mutants but not in *Hk*^2 ^mutants (Table 1C), and similar results were found in wild-type siblings of the same fly lines (Table 1D). A multivariate Cox regression analysis was then performed, to test how the individual's hazard was affected by 1) total sleep, after having corrected for the effects of total activity, and 2) total activity, after having corrected for total sleep. We found that in both *Hk*^1 ^and *Hk*^*Y *^mutants only total sleep during the first 4 weeks of age affected hazard, and in 2 control lines total sleep affected hazard more significantly than did total activity (Table 2). Similar results were also obtained when hazard was measured relative to sleep during the first 2 weeks of age (not shown). Thus, these data suggest that while sleep associates significantly with lifespan variation even after any effects of activity are removed, the converse is not always true.

**Table 2:** Multivariate Cox regression analysis in Hk mutants (A) and their wild-type siblings (B)

A				
Line	Days	Variable	Regression coefficient (×10^2^)	P
HkY	28 d	**Total Sleep**	**-5.20**	**0.002**
	28 d	Total Activity	0.09	0.865
				
Hk1	28 d	**Total Sleep**	**-6.69**	**<0.0001**
	28 d	Total Activity	-0.39	0.442
				
Hk2	28 d	Total Sleep	1.00	0.621
	28 d	Total Activity	1.31	0.260
B				
				
Wt-HkY	28 d	Total Sleep	-0.03	0.989
	28 d	Total Activity	1.20	0.278
				
Wt-Hk1	28 d	**Total Sleep**	**-4.32**	**<0.0001**
	28 d	**Total Activity**	**0.55**	**0.014**
				
Wt-Hk2	28 d	**Total Sleep**	**-5.82**	**0.003**
	28 d	**Total Activity**	**-3.03**	**0.016**
				

## Discussion

In humans it is assumed that a chronic decrease in sleep duration impairs health through the same mechanisms by which acute sleep deprivation and sleep restriction may act, including decreased glucose sensitivity and increased insulin resistance [39,40], increased blood pressure and heart rate (e.g. [39,41], and blunted nocturnal decline in blood pressure [42]. Another potential mechanism is increased metabolic rate, since peripheral metabolic rate is increased in insomniacs relative to normal sleepers, in normal sleepers on nights of poor sleep relative to baseline nights [43], and in patients with fatal familial insomnia [44]. Consistent with human studies, a recent study found that flies selected through repeated genetic crossings for short and fragmented sleep are hyperactive, show increased body lipid content and reduced lifespan [45], but whether short sleep per se (without sleep fragmentation or hyperactivity) can affect longevity could not be tested. Our regression analysis found that both short sleep amount and high wake activity were associated with increased risk of death in 4 of the 6 tested lines. These lines included the 2 most extreme short sleeper mutants (*Hk*^1 ^and *Hk*^*Y*^) as well as their wild-type controls. There was no significant association in the weak hypomorphic *Hk*^2 ^mutants and their wild-type controls, although a trend towards a negative association with sleep amount was present in both mutants and controls (first 28 days, *Hk*^2 ^p = 0.15, controls p = 0.08), while a trend towards a positive association with wake activity was present in mutants only (first 28 days, *Hk*^2 ^p = 0.078). This analysis shows that when both sleep amount and wake activity are used in the same regression, the effects of activity are much reduced, while most of the sleep effects remain significant. Of note, all 6 lines were tested only after repeated outcrossing to wild-type (Canton-S) flies, to control for possible effects of genetic background on lifespan. However, whereas *Hk*^1 ^and *Hk*^*Y *^mutants and siblings were tested simultaneously, *Hk*^2 ^mutants and their controls were tested 4-5 months later, suggesting that perhaps the difference between wild-type siblings of different crosses arises from environmental fluctuations. Overall, these data support the idea that lifespan is affected by many factors, and suggests that in addition to environment and genetic background, sleep may also in some cases affect longevity. Whether the link between sleep and lifespan is causal, however, remains to be determined. Still, we can conclude that, if there is a causal relationship with lifespan, the causal variable is most likely sleep, not activity. Moreover, it is intriguing that *Hk*^1 ^and *Hk*^*Y *^flies were short sleepers especially early in life, while *Hk*^2 ^flies only became short sleepers starting 2 weeks after eclosure. This suggests that the effects of sleep on aging may be especially prominent early in life.

Many studies have found that individuals with disturbed sleep (i.e. who reported either difficulties in falling asleep or regular use of hypnotics) have increased risk of cardiovascular disease [6,46], diabetes [47], and overall mortality [3,48,49]. However, the relative contribution of sleep fragmentation and short sleep duration is difficult to assess. One study specifically tested only individuals with the same total sleep time (~6.5 hours/night) and found that fragmented sleep (>9 microarousals/hour) *per se *was associated with increased levels of lipids, cortisol, and blood pressure [50]. In flies we could not assess the effects of fragmented sleep independently of those of short sleep, because all parameters related to sleep quantity and quality were strongly correlated. It should also be mentioned that brief awakenings are hard to assess in flies, where sleep and waking are usually calculated based on 1-min time bins (microarousals in humans last only a few seconds [51]). This explains why we and others [33] counted only 4-5 microarousals/24 h.

Our results also show that the effects of *Hk *mutations on the sleep phenotype depend not only on genetic background but also on age and environmental conditions. We previously reported that young adult flies (< 2 week old) carrying *Hk*^1 ^and *Hk*^*Y *^mutations are short sleepers, while *Hk*^2 ^mutants have normal amount of sleep [28]. Here, we find that *Hk*^2 ^mutants become short sleepers when they age, while in old *Hk*^1 ^and *Hk*^*Y *^mutants the short sleeping phenotype becomes less prominent. These results were observed no matter whether all flies or only healthy flies that lived at least 73 days were included in the analysis, suggesting that they may reflect a real age-dependent change in the way *Hk *affects sleep.

In healthy humans the circadian arousal signal in the evening becomes weaker with aging, resulting in more sleep during the so-called "wake maintenance zone" [52]. In flies Koh and colleagues found a decrease in the strength of the rest/activity rhythm with aging, i.e. sleep became more distributed over the 24-hour cycle, rather than occurring mostly at night. We also found that older flies sleep more at the light-dark and dark-light transitions, when younger flies are most active. Of note, however, these changes occurred mainly between day 20 and day 45 (at ~ half of lifespan), and did not seem to progress further with age. This lack of progression may reflect a ceiling effect, or the fact that aging modulates night vs day sleep preference only during a specific time window in "middle age". It is also possible, however, that the blunted circadian regulation of sleep is due to factors other than aging per se.

In healthy humans aging is associated with a decrease in total sleep time and in sleep efficiency (the number of awakenings after sleep onset increases), although between elderly (60-70 years) and older elderly (>70) there is no change except in sleep efficiency [32]. One study in flies [20] found that sleep amount in wild-type (Canton-S) females was significantly lower at day 33 than at day 3, consistent with our results in males. Another study [33] found no decrease in total sleep duration with aging in male Canton-S flies maintained at 25°C, and a moderate increase in females [33]. The same study [33] found that the number of brief awakenings and of sleep episodes increased after ~ day 30, while the duration of sleep episodes decreased, at least in females, and the effects were more pronounced at 25°C and 29°C than at 18°C. In 5 of our 6 tested lines, all kept at 20°C, we did not find clear signs of aging-related sleep fragmentation, including the 3 wild-type lines. In fact, if anything, we found a decrease in the number of brief awakenings in old flies relative to young ones, and except in the case of *Hk*^2 ^mutants, no change or an increase in the duration of sleep episodes with aging. The number of sleep episodes did increase mostly at the beginning and at the end of the light period (accounting for the increase in total sleep duration), but only by ~ 10-20%, while Koh and colleagues [33] reported a > 2-fold increase. We also reared and tested *Hk*^1 ^mutants and wild-type siblings at 25°C, but found trends similar to those observed at 20°C, and no clear evidence of sleep fragmentation. It should be stressed, however, that the analysis of brief awakenings in flies (using 1-min bins) may not be sensitive enough to detect subtle changes in sleep quality. Despite this limitation, our results suggest that a decay in sleep quality is not necessarily a consequence of aging in flies.

## Conclusions

These results show that short sleep duration and reduced longevity might be linked in flies, independent of mutations affecting the Shaker current. Moreover, they show that the strong preference for night sleep relative to day sleep is reduced in middle age flies, which have more sleep episodes at the beginning and the end of the light period compared to younger flies. Finally, these data suggest that a decay in sleep quality does not consistently occur in flies with aging, and when it does, it may depend on multiple factors including rearing conditions and genetic background.

## List of abbreviations

CS: Canton-S; *Hk: Hyperkinetic*; *Sh: Shaker*; WT: wild-type.

## Authors' contributions

DB carried out the experiments and drafted the manuscript; KAH performed the analysis of lifespan; GT designed the experiments and helped in the last version of the manuscript; CC designed the experiments, coordinated and supervised the project, and wrote part of the manuscript. All authors read and approved the final manuscript.
